# Association between Bone Mineral Density and Incidence of Breast Cancer

**DOI:** 10.1371/journal.pone.0070980

**Published:** 2013-08-05

**Authors:** Merav Fraenkel, Victor Novack, Yair Liel, Michael Koretz, Ethel Siris, Larry Norton, Tali Shafat, Shraga Shany, David B. Geffen

**Affiliations:** 1 Endocrine Unit, Columbia University Medical Center, New York, New York, United States of America; 2 Clinical Research Center, Columbia University Medical Center, New York, New York, United States of America; 3 Breast Health Center, Columbia University Medical Center, New York, New York, United States of America; 4 Division of Endocrinology, Columbia University Medical Center, New York, New York, United States of America; 5 Breast Center, Memorial Sloan Kettering Cancer Center, New York, New York, United States of America; 6 Department of Clinical Biochemistry, Ben-Gurion University of the Negev, Beer Sheva, Israel; 7 Department of Oncology, Soroka University Medical Center and the Faculty of Health Sciences, Ben-Gurion University of the Negev, Beer Sheva, Israel; Ospedale Pediatrico Bambino Gesu’, Italy

## Abstract

**Introduction:**

Previous studies have suggested an inverse relationship between bone mineral density (BMD) and breast cancer incidence. The primary objective of this study was to assess whether BMD is associated with risk of subsequent breast cancer occurrence in the female population of southern Israel.

**Methods:**

The electronic medical charts of women who underwent BMD at the Soroka Medical Center (SMC) between February 2003 and March 2011 were screened for subsequent breast cancer diagnoses. Women were divided by tertiles of BMD at 3 skeletal sites: lumbar spine (LS, L1–4), total hip (TH) and femoral neck (FN). The incidence of breast cancer was calculated.

**Results:**

Of 15268 women who underwent BMD testing, 86 were subsequently diagnosed with breast cancer. Most women in the study were older than 50 years (94.2% and 92.7%, respectively; p = 0.597). Women who subsequently developed breast cancer had a higher mean body-mass index (BMI) (30.9±5.5 vs. 29.1±5.7 p = 0.004) and the mean BMD Z-score was significantly higher than in those without breast cancer for all 3 skeletal sites (LS: 0.36±1.58 vs. −0.12±1.42, p = 0.002; TH: 0.37±1.08 vs. 0.03±1.02, p = 0.002; FN: 0.04±0.99 vs. −0.18±0.94; p = 0.026). Women in the highest Z-score tertiles at the FN and TH had a higher chance of developing breast cancer compared to the lowest tertile; odds ratio of 2.15, 2.02, respectively (P = 0.004 and 0.01 respectively). No association was found between the BMD Z-score and the stage, histology, grade or survival from breast cancer.

**Conclusions:**

This study provides additional support for an inverse association between BMD and the risk of breast cancer.

## Introduction

The incidence of both osteoporosis and breast cancer increases following menopause [1]. Estrogen has a central role in the maintenance of bone integrity. Its deficiency produces accelerated bone resorption leading to decreased bone mineral density (BMD) [2]. BMD is considered by some to be a marker for lifetime estrogen exposure [3]. The prolonged duration of exposure to estrogen, reflected by reproductive history, young age at menarche, late menopausal age and the use of hormone replacement therapy are known to affect risk of breast cancer [1].

Several observational studies have suggested that a higher bone mass is associated with increased breast cancer risk and less favorable prognosis of an existing breast cancer [2], [4]–[12]. In one of these studies the increase in breast cancer risk in women with higher BMD was shown to be independent of the Gail score, commonly used to evaluate breast cancer risk [5]. The proposed biological explanation for the association of BMD and the risk for breast cancer is via the stimulating effects of estrogen on both bone and mammary cells. In contrast to this, several other studies have shown no association between BMD and the risk for breast cancer in both post- and premenopausal women [13]–[17]. The largest existing case-control study failed to demonstrate an independent association between BMD and risk for breast cancer in postmenopausal women [13].

In view of these conflicting data, we conducted this retrospective study with the primary objective to assess whether BMD is associated with risk of subsequent diagnosis of breast cancer in an unselected population of women of southern Israel.

## Methods

The study protocol was approved by the institutional review board (IRB) at Soroka Ben-Gurion University medical center. This study was based on a retrospective database analysis, therefore the need for an informed consent form was waived by the IRB.

### Population

We retrospectively identified all the women who underwent a BMD measurement at Soroka Medical Center, Beer-Sheva, Israel, a 1000 bed tertiary-care hospital, between February 2003 and March 2011. We excluded those under age 18 and women with a known history of breast cancer at the time of BMD testing. Only the initial test was included in the analysis for women who underwent more than one BMD measurement. The electronic medical charts of the patients were screened for breast cancer diagnosed after BMD testing; using ICD-9 codes (174.0–175.9). Any diagnosis of breast cancer was included, irrespective of grade or stage. The following data were collected: demographic data, mortality data, BMI, laboratory results, and breast cancer characteristics (histological diagnosis, stage and grade).

### BMD Measurement

Bone mineral density was measured by dual energy X-ray absorptiometry (DEXA) using a Prodigy densitometer (GE-Lunar, Milwaukee, USA).

Bone mineral density was assessed at the lumbar spine (LS), femoral neck (FN) andtotal hip (TH) and was expressed as either bone density (in g/cm^2^), T-score (standard deviation from the mean for young women) or Z-score (standard deviation from the mean for age-matched women adjusted for body mass).Z-score and T-score were calculated according to the Lunar-Prodigy manufacturer’s internal database and, based on NHANES III data.

### 25 OH Vitamin D Assay

Vitamin D status was reported when available. Vitamin D levels were determined by measuring patients’ serum 25-hydroxyvitamin D (25(OH)D) levels by the IDS Octavia 25-OH-D kit (Immunodiagnostic Systems, Boldon, UK). Results were expressed as ng/ml, normal range: 20–58 ng/ml.

### PTH Assay

PTH status was reported when available. Serum PTH levels were determined by using the Immulite 2000 intact PTH kit (Siemens, Los Angeles, CA. USA). This determination is based on a solid phase, two-site chemiluminescent enzyme – labeled immunometric assay. Results were expressed as pg/ml; normal range: 14–72 pg/ml.

### Primary Endpoint

To assess the association between BMD and breast cancer, we defined the primary endpoint as the incidence of new breast cancer diagnosis (consistent with the ICD-9 codes) recorded after the BMD testing date.

### Statistical Analysis

The results are presented as the mean ± SD for continuous variables and as the total patients (percentage of total patients) for categorical data. T-test and one-way ANOVA were used for comparison of the continuous variables and chi-square or Fisher’s exact tests for categorical data. We used Mann-Whitney test for the comparison of variables with non-normal distribution. Correlations between BMD and breast cancer stage and grade were assessed by Spearman’s rank correlation coefficient. Time to diagnosis of breast cancer and survival analyses among breast cancer patients, were calculated by Kaplan-Meier survival analysis and the log-rank test. Multivariate analyses were performed using Cox regression, where BMD divided into tertiles was adjusted for age and BMI.A two-tailed *P*-value of ≤0.05 was considered significant. The statistical analysis was done using SPSS version 18.

## Results

### Study Population

**Figure 1:**
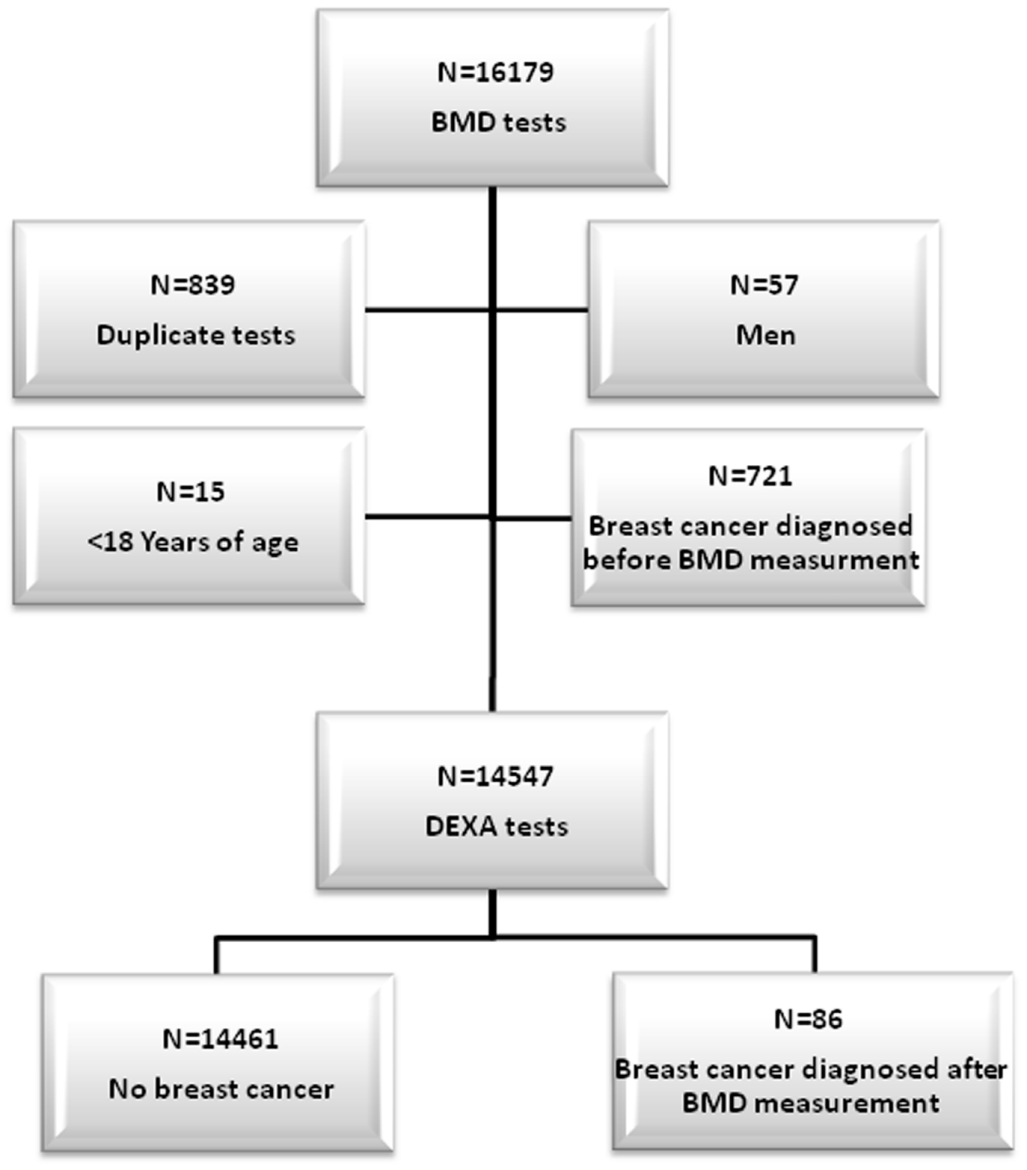
Flow chart of the study population selection.

Between February 2003 and March 2011 a total of 16,179 BMD tests were performed at the Soroka University Medical Center. Figure 1 presents the study population flow chart. We excluded 911 tests (57 men, 839 repeat BMD tests, and 15 children under the age of 18). Furthermore, 721 women had a breast cancer diagnosis prior to the DEXA BMD measurement, leaving14,461 women who had no prior diagnosis of breast cancer at the time of the BMD measurement.

During a median follow up of 47 months (inter-quartile range of 26–66 months) following BMD measurement, 86 women were diagnosed with breast cancer. For the present study, we compared the 86 women who developed breast cancer following BMD measurement with 14,461 who remained cancer free.

Table 1 presents the baseline characteristics of the patient population. A similar percentage in both groups were above the age of 50 years; 94.2% vs. 92.7% p = 0.60. However, women who were diagnosed with breast cancer were on average 3.5 years older than those who remained cancer-free. Women who developed breast cancer also had a slightly higher BMI than those who remained cancer-free. Serum levels of 25(OH) D and PTH (available, respectively, for 44% and 17% of women who developed breast cancer, and for 36% and 14% in the group of women who did not develop breast cancer) were not statistically different between the two groups.

**Table 1:** Tested population characteristics at the time of BMD measurement. Age, BMI25(OH)D and PTH are presented as mean ± SD.

Variable	Breast cancer (n = 86)	No breast cancer (n = 14461)	P value
Age	68.78±9.12	65.12(±11.01)	<0.001
Age ≥50	81 (94.2)	13404 (92.7)	0.597
BMI(kg/m^2^)	30.89±5.46	29.11±5.67	0.004
25-(OH) vitamin D (ng/ml)*	18.97±9.33	19.95±8.99	0.505
PTH (pg/ml)**	99.85±88.49	71.89±141.45	0.445

* Data was available fort 40% of the women.

** Data was available for 15% of the women.

### Bone Mineral Density

Mean BMD was somewhat higher, at all skeletal sites, in women who developed breast cancer compared to those who remained cancer-free (Table 2). Mean BMD T-score was higher in women who were subsequently diagnosed with breast cancer reaching statistical significance only for the total hip. Mean BMD Z-score was significantly higher at all skeletal sites in women diagnosed with breast cancer.

**Table 2:** BMD results at 3 skeletal sites in women with and without subsequent diagnosis of breast cancer.

Site	Variable	Breast cancer n = 86; (mean ± SD)	No breast cancer n = 14461 (mean ± SD)	P value
Femoral Neck	BMD (g/cm^2^)	0.84±0.13	0.81±0.13	0.12
	T-score	−1.21±1.12	−1.39±1.07	0.12
	Z-score	0.04±0.99	−0.18±0.94	0.026
Total hip	BMD (g/cm^2^)	0.91±0.15	0.88±0.14	0.033
	T-score	−0.74±1.24	−1.02±1.16	0.03
	Z-score	0.37±1.08	0.03±1.02	0.002
Spine (L1–4)	BMD (g/cm^2^)	1.04±0.19	1.00±0.17	0.056
	T-score	−1.15±1.6	−1.46±1.44	0.055
	Z-score	0.36±1.58	−0.12±1.42	0.002

**Figure 2:**
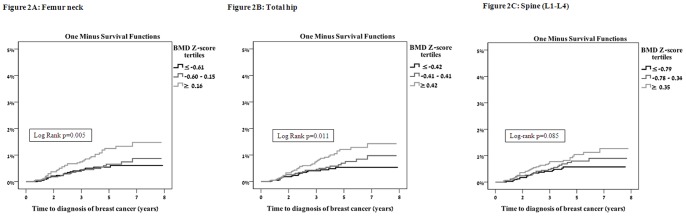
Time to diagnosis of breast cancer following BMD measurement in tertiles of BMD Z score at three skeletal sites. A. Femoral neck, B. Total hip, C. Lumbar spine.

BMD results were divided into 3 cohorts based on tertiles of Z-score. Figure 2 depicts Kaplan Meier curves of breast cancer incidence following BMD testing. For FN and TH there was a statistically significant increased incidence of breast cancer in women in the highest compared to the lowest tertile of Z-score BMD. A similar trend, which did not reach statistical significance, was observed regarding LS Z-score.

Table 3 presents the results of Cox proportional hazards regression models. Comparing the highest with the lowest tertile for BMD Z-score at the FN and TH, the increased risk for breast cancer translated to hazard ratios of 2.15 (P = 0.004) and 2.05 (P = 0.01), respectively. Comparing the highest with the lowest Z-score BMD at the LS revealed a similar trend, which did not reach statistical significance.

**Table 3:** Hazard ratio for acquiring breast cancer diagnosis according to BMD Z-score tertiles.

Site	Tertile	Hazard ratio	95% CI	P value
Femoral neck	1st tertile	1		
	2nd tertile	1.15	0.64–2.08	0.644
	3rd tertile	2.15	1.27–3.64	0.004
Total hip	1st tertile	1		
	2nd tertile	1.34	0.74–2.43	0.328
	3rd tertile	2.05	1.19–3.54	0.01
Spine (L1–44)	1st tertile	1		
	2nd tertile	1.29	0.71–2.33	0.404
	3rd tertile	1.64	0.93–2.89	0.087

The lowest Z- score tertile was used as reference (HR = 1).

Multivariate analysis (Cox regression) adjusted for age and BMI.

### Stage, Grade and Survival from Breast Cancer

Among the 86 women diagnosed with breast cancer following BMD testing, we did not find any association between BMD at any of the skeletal sites and breast cancer stage, histology or grade (Table 4). ER receptor status was available for 63 of 86 women with breast cancer and 86% of these were found to be ER positive. At 48 months follow up, there were 11 death events in the breast cancer group (seven deaths were breast cancer related, and four were unrelated to the disease) with no apparent association between BMD and survival.

**Table 4:** Breast cancer characteristics according to femur neck BMD Z score tertiles (N = 86).

Variable	First tertile (< = −0.47)	Second tertile (−0.46 − 0.34)	Third tertile (> = 0.35)	P value
Grade (median, inter-quartile range)	1−(1–2)	2 (1–3)	2 (2–3)	0.292
T stage (median, inter-quartile range)	1 (1–2)	1 (1–2)	1 (1–2)	0.888
N stage (median, inter-quartile range)	0 (0–1)	0 (0–1)	0 (0–1)	0.510
Survival at 5 years (KM rate)	69%	73%	83%	0.523

This paper was presented in part at the annual meeting of the Endocrine Society, Houston Texas USA Jun 2012.

## Discussion

This is the first study reporting on the association between bone mineral density and the risk of breast cancer in an Israeli population. Our results show that women who subsequently developed breast cancer had a higher baseline BMD at all three skeletal sites (lumbar-spine, femoral neck and total hip) compared with those who remained free of breast cancer during the follow-up period. Furthermore, higher BMD Z-scores at the femoral neck and total hip maintained the association with an increased risk of incident breast cancer, even after adjustment for age and BMI.

Our results are in line with several previously reported prospective and retrospective studies which revealed higher incidence of breast cancer in postmenopausal woman with higher BMD [2], [4]–[13], [15]–[17]. The majority of the reports originate from large databases such as the Rotterdam [6], Women’s Health Initiative (WHI) [5] and Framingham [16] studies or from pivotal trials in the field of osteoporosis (SOF, FIT, DOES) [2], [7], [9], that integrated data on BMD and breast cancer incidence in the same patient (2,4–7,9). Other studies supporting the association of higher BMD with greater risk of breast cancer derived from local BMD registries that were related to breast cancer registries in the same area [8], [10]. In some of these reports BMD was shown to be associated with increased breast cancer risk after adjusting for variables representing life-time estrogen exposure (lifetime ovulation period, BMI etc.) [2], [6], [7] One of those studies demonstrated that the contribution of BMD to the prediction of incident postmenopausal breast cancer was independent of the Gail score, a breast cancer risk-assessment tool [5]. A French prospective study revealed a negative association between higher BMD tertiles and survival from breast cancer [8].

The largest cohort, reported by Grenier et al. and originating from Manitoba, Canada, consisted of 37,860 woman 50 years and older who were followed for a median of 5.4 years after BMD measurement. The study revealed an increased breast cancer risk for the third and fourth quartiles of lumbar spine BMD and for the third quartile for femoral neck BMD after adjusting for age and HRT use [10].

Hadji et al reported the results of MABOT I and II prospective trials that found higher bone mineral density both by quantitative ultrasound and DEXA measurements in new breast cancer patients compared to controls [11], [12].These differences remained significant even after post-matching for possible confounding variables such as age BMI and estrogen exposure.

A number of studies have challenged the existence of association between higher BMD and increased breast cancer risk both in peri-menopausal and early menopausal women [16]–[17]. Others have shown that the positive relationship between BMD and breast cancer risk can be largely explained by effects of covariates and endogenous hormones such as age at menarche and menopause [13], [15]. In one study, an association between higher BMD and the risk for breast cancer was only observed in women with low estradiol level [15].

These studies showing no association between BMD and future breast cancer raise the possibility that BMD may reflect lifetime estrogen exposure rather than being an independent risk factor for future breast cancer risk. Estrogen exposure, as reflected by age at menarche and menopause, gravidity, parity and use of postmenopausal HRT is an established risk factor for breast cancer [1]. At the same time it is well established that higher estrogen exposure is beneficial for bone mass [2]. Therefore the association between BMD and breast cancer risk can be confounded by the lifetime estrogen exposure. This hypothesis is supported by a case control study comparing BMD in 79 postmenopausal women recently diagnosed with breast cancer to 158 age-matched control women with normal mammograms, revealing no differences in BMD between the breast cancer patients and the control group [14]. The authors concluded that BMD should not be used as a pre-screening predictor of mammography results in older women, as has been suggested by others [18]. This was a rather small study that was the first to challenge the common paradigm of higher BMD conferring higher risk for breast cancer. It is conceivable that performing a similar study on a larger cohort may even reverse the relationship possibly showing that breast cancer patients may have lower BMD secondary to release of cytokines by the tumor.

Vitamin D may be an additional common confounder for the association of breast cancer with higher BMD, as higher levels of 25(OH)D are associated with higher BMD [19], improved bone health and lower breast cancer as previously suggested [20]. Information on 25(OH)D levels close to the time of BMD measurement were available in 44% and 36% of breast cancer patients and those without breast cancer, respectively, and did not differ between the two groups. Thus, we cannot draw any conclusion on the relevance of vitamin D levels on the association between breast cancer and BMD.

Our retrospective study is the first to show a positive association of higher BMD with higher incidence of breast cancer in an Israeli population, which is a mix of both Ashkenazi and Sephardi Jews and Arabs. Although several studies have suggested a relationship of the type we observed, others have not. The conclusions that have been reached in this unique population are important for Israel and also support the studies that showed higher rates of incident breast cancer in women with higher BMD.

One limitation of our study is the lack of data regarding parameters of estrogen exposure (gravidity, parity, previous use of birth control pills, use of HRT and age at menarche and menopause). According to the NCI website, the 5 year risk for breast cancer of a woman with an age similar to the mean age of our group (67 years), is between 1.1–2.1%, while the rate of breast cancer in our cohort was lower 0.6%, which is also low compared to that reported in other studies [21]. This most probably is due to the short follow up period (mean 47 months). In addition, we cannot exclude that a small number of women had a breast cancer not accounted for in the computerized patients’ files. Finally, only a fraction of the patients had vitamin D and PTH measurements available.

The study strength is based on the unique structure of Israeli national health-insurance system which includes the entire population and a fully computerized medical record system which maximizes availability of baseline and follow-up data from cradle to grave.

In conclusion, our retrospective study demonstrates a positive correlation between BMD and breast cancer incidence in the population of southern Israel. In view of the contradictory data on the association of BMD and breast cancer risk, a large prospective trial is required, which may allow a further characterization of this association and shed light on possible confounders.
