# Correction: Association between Bone Mineral Density and Incidence of Breast Cancer

**DOI:** 10.1371/annotation/2811f3e3-8efe-4183-bcd7-e4a11401fb89

**Published:** 2013-09-04

**Authors:** Merav Fraenkel, Victor Novack, Yair Liel, Michael Koretz, Ethel Siris, Larry Norton, Tali Shafat, Shraga Shany, David B. Geffen

There were errors in the author affiliations. The correct affiliations are available below.

Merav Fraenkel^1*^, Victor Novack^2^, Yair Liel^1^, Michael Koretz^3^, Ethel Siris^4^, Larry Norton^5^, Tali Shafat^2^, Shraga Shany^6^, David B. Geffen^7^

1 Endocrine Unit, Soroka University Medical Center and the Faculty of Health Sciences, Ben-Gurion University of the Negev, Beer Sheva, Israel,

2 Clinical Research Center, Soroka University Medical Center and the Faculty of Health Sciences, Ben-Gurion University of the Negev, Beer Sheva, Israel

3 Breast Health Center, Soroka University Medical Center and the Faculty of Health Sciences, Ben-Gurion University of the Negev, Beer Sheva, Israel

4 Division of Endocrinology, Columbia University Medical Center, New York, New York, United States of America,

5 Breast Center, Memorial Sloan Kettering Cancer Center, New York, New York, United States of America,

6 Department of Clinical Biochemistry, Ben-Gurion University of the Negev, Beer Sheva, Israel,

7 Department of Oncology, Soroka University Medical Center and the Faculty of Health Sciences, Ben-Gurion University of the Negev, Beer Sheva, Israel 

